# The impact of frailty on clinical outcomes of older patients undergoing enhanced recovery after lumbar fusion surgery: a prospective cohort study

**DOI:** 10.1097/JS9.0000000000001594

**Published:** 2024-05-09

**Authors:** Shuai-Kang Wang, Qi-Jun Wang, Peng Wang, Xiang-Yu Li, Peng Cui, Dong-Fan Wang, Xiao-Long Chen, Chao Kong, Shi-Bao Lu

**Affiliations:** a Department of Orthopedics, Xuanwu Hospital, Capital Medical University; b National Clinical Research Center for Geriatric Diseases, Beijing, People’s Republic of China

**Keywords:** enhanced recovery after surgery, frailty, lumbar fusion, oldest-old patients, risk factors

## Abstract

**Background:**

Frailty is recognized as a surrogate for physiological age and has been established as a valid and independent predictor of postoperative morbidity, mortality, and complications. Enhanced recovery after surgery (ERAS) can enhance surgical safety by minimizing stress responses in frail patients, enabling surgeons to discharge patients earlier. However, the question of whether and to what extent the frailty impacts the post-ERAS outcomes in older patients remains.

**Materials and methods:**

An evidence-based ERAS program was implemented in our center from January 2019. This is a prospective cohort study of patients aged ≥75 years who underwent open transforaminal lumbar interbody fusion (TLIF) for degenerative spine disease from April 2019 to October 2021. Frailty was assessed with the Fried frailty scale (FP scale), and patients were categorized as non/prefrail (FP 0-2) or frail (FP ≥ 3). The preoperative variables, operative data, postoperative outcomes, and follow-up information were compared between the two groups. Univariate and multivariate logistic regression analyses were used to identify risk factors for 90-day major complications and prolonged length of hospital stay after surgery.

**Results:**

A total of 245 patients (age of 79.8±3.4 year) who had a preoperative FP score recorded and underwent scheduled TLIF surgery were included in the final analysis. Comparisons between nonfrail and prefrail/frail patients revealed no significant difference in age, sex, and surgery-related variables. Even after adjusting for multiple comparisons, the association between Fried frailty and ADL-dependency, IADL-dependency, and malnutrition remained significant. Preoperative frailty was associated with increased rates of postoperative adverse events. A higher CCI grade was an independent predictor for 90-day major complications, while Fried frailty and MNA-SF scores <12 were predictive of poor postoperative recovery.

**Conclusion:**

Frail older patients had more adverse post-ERAS outcomes after TLIF compared to non/prefrail older patients. Continued research and multidisciplinary collaboration will be essential to refine and optimize protocols for surgical care in frail older adults.

## Introduction

Highlights

- This is the first study to correlate the Fried frailty with poor postoperative recovery after lumbar fusion surgery.
- The association between Fried frailty and impaired activities of daily living, impaired instrumental activities of daily living-dependency, and malnutrition remained significant even after adjusting for multiple comparisons.
- Fried frailty was associated with increased major adverse events rates following scheduled transforaminal lumbar interbody fusion surgery in postenhanced recovery after surgery patients aged ≥75 years.
- Fried frailty and malnutrition were independent predictors for poor postoperative recovery.

Degenerative lumbar disorders involve a spectrum of pathology, including lumbar intervertebral disk degeneration, facet joint degeneration, ligamentum flavum thickening, and hyperosteogeny, often resulting in low back pain and/or radiculopathy^1^. The burden of lumbar spinal disorders has increased substantially with the unprecedented aging population and the increase in life expectancy, affecting about 40% of older adults aged 65 years and older in the US^2–4^. Surgical fusion has become one of the most performed procedures for degenerative diseases of the lumbar spine. In 2015, the hospital costs for elective lumbar degenerative fusions exceeded $10 billion, the highest aggregate costs of any surgical procedure in the US^5^. Recent studies using multicenter data have shown that the increase in spinal fusion for lumbar spinal degenerative diseases was highest among patients aged over 75 years^6,7^. Although lumbar fusion surgery can effectively alleviate pain-related symptoms and improve quality of life, the high incidence of postoperative adverse outcomes in older patients remains a global challenge^8^. Enhanced recovery after surgery (ERAS) is a multidisciplinary, perioperative management pathway that aims to reduce surgery-related stress response and accelerate postoperative rehabilitation. Since Kehlet and Wilmore first proposed the concept of fast-track surgery, ERAS has been successfully applied in many surgical fields, such as gastrointestinal, hepatobiliary, and pancreatic surgery, and ERAS has achieved considerable results^9^. Evidence-based standardization of perioperative management of lumbar fusion patients by implementing ERAS protocols can lead to improved outcomes, including shorter hospital stay, fewer complications, and lower mortality rates^10^. Nevertheless, age remains a significant risk factor for postoperative adverse events (AEs) even with the implementation of ERAS protocol (Post-ERAS), possibly due to the lower adherence to ERAS regimens, diminished physiological reserves, and potential impairment of organ functions^11,12^.

In addition to the comorbid risk factors that older patients share with younger ones, older patients may also have malnutrition and cognitive or functional impairment, which manifest as frailty. Frailty is the constellation of generalized organ and tissue atrophy, reduced physiological, physical, and cognitive reserves, deconditioning, sarcopenia, and malnutrition^13^. Symptomatic lumbar degenerative disorders are associated with a high prevalence and/or incidence of frailty in community-dwelling elderly persons. In a case–control study, approximately half of the participants with lumbar spinal disorders were frail^14^. Recognized as a surrogate for physiological age, frailty has been established as a valid and independent predictor of postoperative morbidity, mortality, and complications. Two of the most common instruments to assess frailty are the frailty index (FI)^15^ and the frailty phenotype (FP) developed by Fried *et al*.^16^. The FP scale is a validated instrument that captures the physiological and functional decline of the body over time, and it is extensively utilized in clinical practice for frailty screening. Fried frailty criteria include more objective and modifiable items (e.g. body weight and grip strength) than other frailty assessment instruments. Therefore, the Fried FP might be the most appropriate instrument for guiding antifrailty treatment (e.g. multicomponent exercise and prerehabilitation).

ERAS can enhance surgical safety by minimizing stress responses in frail patients, enabling surgeons to discharge patients earlier^10,17^. However, the question of whether and to what extent the frailty impacts the post-ERAS outcomes in older patients remains. The purpose of this study was to evaluate the impact of frailty, as determined by the FP scale, on post-ERAS outcomes, including length of hospital stay (LOS), nonhome discharge, and 90-day readmissions in older patients (aged 75 years and above) undergoing lumbar fusion surgery.

## Materials and methods

### Participants

The protocol for this prospective cohort study was registered as a Chinese clinical trial and received approval from the regional medical ethics committee (IRB#2018-086). The work has been reported in line with the strengthening the reporting of cohort, cross-sectional, and case–control studies in surgery (STROCSS) criteria^18^. Patients aged ≥75 years who were scheduled to undergo lumbar fusion surgery for degenerative spine disease were included in the study. Patients with fractures, spine metastasis, and spinal infections were excluded from the study. Patients who were admitted from the emergency department for an emergency operation were also excluded. Written consent was obtained from all participants. All participants were enrolled from April 2019 to October 2021 and were followed for at least 2 years. After geriatric and surgical risk assessment, all participants underwent open transforaminal lumbar interbody fusion (TLIF) performed by the same surgical team.

### ERAS program

**Figure 1:**
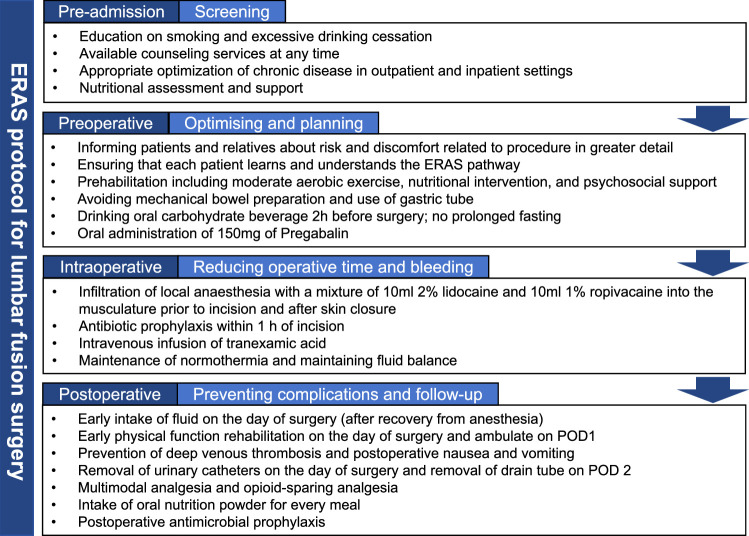
Enhanced recovery after surgery protocol in our center.

The ERAS program consisted of preadmission and perioperative multimodal management and was implemented by the orthopedic department in January 2019. Our ERAS program includes preadmission education and consultation, risk screening and optimization, multimodal analgesia, minimal intravenous fluid administration, pre-emptive analgesia, early removal of urinary tube, early physical rehabilitation, avoiding mechanical bowel preparation, no prolonged fasting, nutritional management, and antithrombotic prophylaxis (Fig.1). The detailed pathway of ERAS has been previously reported^19^.

### Data collection

All data were extracted from the medical record and the electronic inpatients medical record system. Preoperative baseline data included demographic characteristics, smoking status, drinking status, primary diagnosis, duration of symptoms (based on self-reported duration of symptoms prior to surgical intervention), Charlson comorbidity index (CCI), geriatric assessment results, pain levels [measured by visual analog scale (VAS) and Oswestry Disability Index]. Preoperative smoking status was classified as ‘never smoking’, ‘former smoking (stopping smoking for more than 1 year)’, and ‘current smoking’. Drinking status was classified into three groups: never drinking, quit drinking (more than 1 year), and current drinking. Surgery-related variables were also reviewed and collected. Preoperative geriatric assessment included frailty (Fried frailty phenotype)^16^, activities of daily living (ADL)^20^, instrumental ADL (IADL)^21^, mini-mental state examination^22^, and mini-nutritional assessment-short form (MNA-SF)^23^. Considering the difference in the body size between Western and Asian populations, frailty was assessed using the frailty phenotype by Fried and colleagues, with the criteria for the FP adapted from Wu’s research on frailty within the Chinese population^24^. The thresholds for assessing cognition by education level have been defined as follows: illiterate, >17 points; primary school, >20 points; and junior high school and above, >24 points^25^. A description of preoperative assessment tools and their corresponding cutoff values are displayed in Table 1. All patients were evaluated by a multidisciplinary team that consisted of surgeons, geriatrics, neurology, pharmacy, nutrition, nursing, and rehabilitation. Preoperative assessment was conducted within 2 days after admission.

**Table 1:** Description of preoperative assessment tools and their corresponding cut-off values.

Tools	Description of the assessment scales	Cut-off values
Fried frailty phenotype	5-item tool to assess physical frailty phenotype involving five criteria: weight loss, exhaustion, low physical activity, slowness, and weakness	1–2 points for prefrailty; 3–5 points for frailty
Activities of daily living (ADL)	10-item tool to assess basic activities of daily living	≤20 points for extremely severe; 25–45 points for severe; 50–70 points for moderate; 75–95 points for mild dependency (0–100)
Instrumental activities of daily living (IADL)	8-item tool to assess complex activities of daily living needed to live independently	≤2 points for extremely severe; 3 points for severe; 4 or 5 points for moderate dependency; 6 or 7 points for mild dependency (0–8)
Mini-nutritional assessment-short form	6-item tool to identify patients with malnutrition or at risk of malnutrition	<12 points (0–14 points)
Mini-mental state examination (MMSE)	11-item test that includes registration, attention and calculation, recall, language, and orientation	24 points (junior high school and above) 20 points (primary school) 17 points (illiterate)

### Outcome measures

The primary outcome measure was the incidence of overall postoperative AEs including prolonged LOS, nonhome discharge, unplanned readmission, and postoperative complications within 90 days. The discharge criteria for all patients were as follows: (1) preoperative symptoms were entirely or mainly relieved, or treatment met the patient’s expectations; (2) patients had no surgery-related complications, or the postoperative complications had been controlled, and (3) no further treatment was required. The LOS was recorded routinely by hospital administrative staff unaware of the study, and extracted from the hospital’s electronic patient record by a research nurse. Prolonged LOS was defined as postoperative hospital stay longer than the 75th percentile. Nonhome discharge was categorized as discharge to a nursing home/long-term care facility, or rehabilitation center. Other AEs were recorded by an unblinded research nurse using predefined criteria for the presence or absence of 90-day complications and readmission according to the clinical record, medication record, and follow-up data. Postoperative complications were graded by using the Clavien–Dindo grading system for the classification of postoperative adverse outcomes. Clavien–Dindo I–II complications were defined as minor complications, while Clavien–Dindo III–IV complications were defined as major complications^26^. Secondary outcome measures were the incidences of major AEs and minor AEs.

### Statistical analysis

Before patient enrollment, a sample-size calculation was performed using the Pwr package (R Project for Statistical Computing Version 4.3.2, Base Package). The sample size requirement for this trial was calculated based on the following historical data and assumptions. The primary outcome of our study was the incidence of postoperative AEs. Previous studies showed that the AEs rates for frail patients and nonfrail/prefrail patients with lumbar fusion surgery are about 35 and 19%, respectively^27,28^. Based on findings from previous literature and the observed positive correlation between advanced age and frailty, we anticipated that 50% of eligible older patients would exhibit frailty^14^. The minimum sample size of 238 was calculated as an adequate sample required to perform the study, assuming a significance level of 95% (α=0.05) and statistical power (1–β) of 80%. To allow for a 5% dropout rate, 250 patients were planned for inclusion.

Normality testing was performed by the Shapiro–Wilk test of normality and normal Q–Q plots. Normally and non-normally distributed data were presented as mean ± SD and median (quartile 1, quartile 3), respectively. Two-group comparisons were performed using the *t*-test for independent normally-distributed data, Wilcoxon test for dependent not-normally-distributed data, and Mann–Whitney *U* test for independent not-normally-distributed data. Categorical variables were expressed as frequencies with percentages and analyzed using Fisher’s exact and *χ* ^2^ tests, as appropriate. The primary outcome of our study was the incidence of overall AEs. The secondary outcomes were each component of the primary efficacy outcome, including major and minor AEs. A post-hoc Bonferroni calculation was made for reference purposes to derive an adjusted threshold for *P*-values to account for multiple comparisons of secondary outcomes. Factors with a *P*-value of less than 0.1 in the univariate analysis were entered in the multivariate logistic regression, and *P*-values of less than 0.05 were considered to indicate statistical significance. The package of *Corrplot* in R was used for the correlation analysis based on Spearman’s correlation method. Subsequently, *P*-values were adjusted using Bonferroni correction. All analyses were completed using R version 4.3.2 and SPSS version 24 (SPSS, Version 24; IBM).

## Results

**Figure 2:**
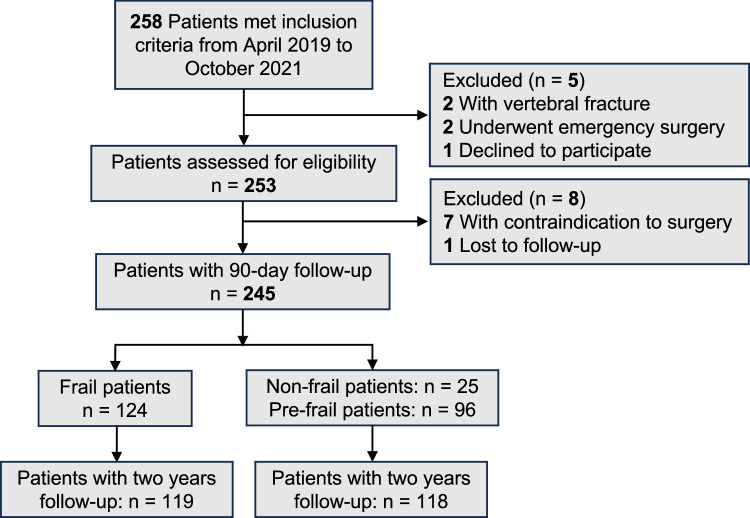
Flow chart describing of the inclusion of individuals.

**Figure 3:**
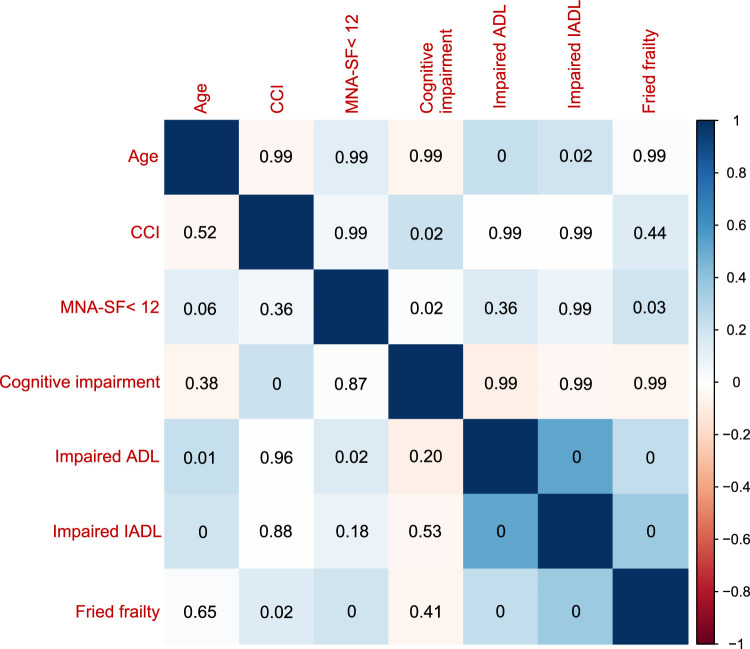
Correlations among the geriatric assessment tools in this study. Red indicates a negative correlation, and blue indicates a positive correlation. The darker the color meant the stronger correlation between the two indicators, while the lighter meant the weaker correlation. Bonferroni corrected *P*-values shown in upper right triangle; uncorrected *P*-values shown in lower left triangle. ADL, activities of daily living; CCI, Charlson comorbidity index; IADL, instrumental activities of daily living; MNA-SF, mini-nutritional assessment-short form index; MMSE, mini-mental state examination.

**Figure 4:**
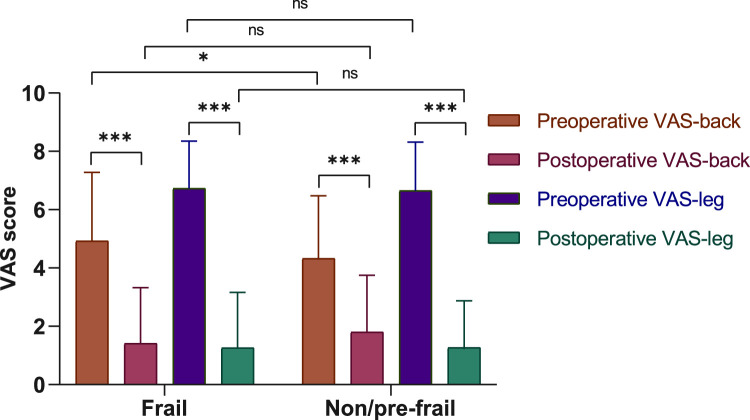
Comparison of preoperative and postoperative visual analog scale scores between non/prefrail and frail group. VAS, visual analog scale.

From April 2019 to October 2021, 245 patients had a preoperative FP score and a subsequent TLIF surgery for lumbar degenerative disorders (Fig. 2). The mean age of patients was 79.8±3.4, and 62.5% were female. Based on previous work, FP scores were categorized as nonfrail (0 points), prefrail (1–2 points), and frail (3–5 points). In this study cohort, 25 individuals (10.2%) were categorized as nonfrail (score 0), 96 (39.2%) as prefrail (score 1–2), and 124 (50.6%) as frail (score ≥3). Due to the data distribution, all scores were dichotomized with a cutoff at 3 into either ‘nonfrail/prefrail’ or ‘frail’. Comparisons between nonfrail and prefrail/frail patients revealed no significant difference in age, sex, and surgery-related variables. There were no significant differences in smoking and drinking status, primary diagnosis, or symptom duration between nonfrail and prefrail/frail patients (Table 2). The correlation matrix in Figure 3 summarizes the correlations among the geriatric assessment tools. Even after adjusting for multiple comparisons, the association between Fried frailty and ADL-dependency, IADL-dependency, and malnutrition remained significant (*P*<0.05). Regarding postoperative outcomes, there were significantly higher rates of overall AEs (52.4 vs. 34.7%, *P*=0.005), major AEs (17.7 vs. 7.4%, *P*=0.016), minor AEs (48.4 vs. 31.4%, *P*=0.007) among frail patients. At the time of the final follow-up, patients in both groups saw significant improvement in patient-reported outcomes compared to the preoperative state (Fig. 4) (Table 3).

**Table 2:** Comparison of patient characteristics and surgery-related data between nonfrail/prefrail and frail patients.

Variable	Total	Nonfrail/prefrail	Frail	*P*
No. of patients	245	121	124	
Female *n*/(%)	153 (62.5%)	74 (61.2%)	79 (63.7%)	0.680
Age (years)	79.8±3.4	79.7±3.2	79.9±3.6	0.712
BMI (kg/m^2^)	24.8±3.6	24.6±3.4	25.0±3.7	0.338
Charlson comorbidity index				0.010*
0–1	148 (60.4%)	83 (66.1%)	65 (52.4%)	
2 or more	97 (39.6%)	38 (33.9%)	59 (47.6%)	
Current smoking	23 (9.4%)	12 (9.9%)	11 (8.9%)	0.779
Current drinking	14 (5.7%)	5 (4.1%)	9 (7.2%)	0.292
Dependence in ADL				<0.001*
No	44 (17.9%)	31 (25.6%)	13 (10.5%)	
Mild	140 (57.2%)	73 (60.3%)	67 (54.0%)	
Moderate-severe	61 (24.9%)	17 (14.1%)	44 (35.5%)	
Dependence in IADL				<0.001*
No	51 (20.8%)	41 (33.9%)	10 (8.1%)	
Mild	70 (28.6%)	37 (30.6%)	33 (26.6%)	
Moderate-severe	124 (50.6%)	43 (35.5%)	81 (65.3%)	
MNA-SF < 12 points	63 (25.7%)	18 (14.9%)	45 (36.3%)	<0.001*
MMSE< 24 points	173 (70.6%)	87 (71.9%)	86 (69.4%)	0.662
*Primary diagnosis*				0.307
LSS	146 (59.6%)	76 (62.8%)	70 (56.5%)	
DDD	59 (24.1%)	24 (19.8%)	35 (28.2%)	
Lumbar spondylolisthesis	40 (16.3%)	21 (17.4%)	19 (15.3%)	
Duration of symptoms (years)	5.9 ± 7.3	6.3 ± 7.8	5.6 ± 6.9	0.403
VAS-back at baseline	4.6 ± 2.3	4.3 ± 2.2	4.9 ± 2.4	0.037*
VAS-leg at baseline	6.7 ± 1.7	6.6 ± 1.7	6.7 ± 1.6	0.701
ODI at baseline	48.6 ± 18.2	47.3 ± 18.4	49.8 ± 18.1	0.295
*Surgery-related data*				
ASA	2.7 ± 0.5	2.7 ± 0.5	2.8 ± 0.5	0.311
Number of fused segments				0.461
1-2	177 (72.2%)	90 (74.4%)	87 (70.2%)	
3-5	68 (27.8%)	31 (25.6%)	37 (29.8%)	
Operative time (min)				
1-2 level fusion	195.8 ± 58.9	192.2 ± 64.2	199.5 ± 53.0	0.413
3-5 level fusion	276.9 ± 74.3	282.5 ± 81.3	272.3 ± 68.6	0.576
Estimated blood loss (ml)	367.6 ± 354.5	363.7 ± 330.9	371.3 ± 377.4	0.868

ADL, activities of daily living; ASA, American Society of Anesthesiologists; DDD, degenerative disc disease; IADL, instrumental activities of daily living; LSS, lumbar spine stenosis; MMSE, mini-mental state examination; MNA-SF, mini-nutritional assessment-short form; ODI, Oswestry Dability Index; VAS, visual analog scale.

* Represents for statistically different (*P*<0.05).

**Table 3:** Postoperative outcomes of patients in nonfrail/prefrail and frail groups.

Variables	Nonfrail/prefrail	Frail	*P*
No. of patients	121	124	
Primary outcomes			
Total AEs	42 (34.7%)	65 (52.4%)	0.005
Secondary outcomes			
Major AEs	9 (7.4%)	22 (17.7%)	0.015*
Major complications	9 (7.4%)	21 (16.9%)	
Unplanned readmission	4 (3.3%)	10 (8.1%)	
Reoperation	5 (4.1%)	2 (1.6%)	
Minor AEs	38 (31.4%)	60 (48.4%)	0.007*
Prolonged LOS	21 (17.4%)	40 (32.3%)	
Minor complications	21 (17.4%)	24 (19.4%)	
Nonhome discharge	5 (4.1%)	14 (11.3%)	
LOS (day)	14 (12,18)	16 (13,21)	

AEs, adverse events; LOS, length of hospital stay.

* Post-hoc correction for multiple testing of two secondary outcomes by the Bonferroni method resulted in a *P*-value of 0.025 to indicate a significant difference between groups.

Univariate and multivariate analysis identified multiple risk factors for prolonged LOS and 90-day major complications. Univariate analysis showed that frailty and greater CCI grade were significantly associated with 90-day major complications; however, multivariate logistic regression analysis revealed that greater CCI grade [odd ratio (OR) 2.29; 95% CI: 1.04-5.08] was the only independent risk factor for major complications (Table 4). Univariate and multivariate analysis indicated that Fried frailty (OR 1.97; 95% CI: 1.03–3.78, *P*=0.041), MNA-SF <12 points (OR 1.94; 95% CI: 1.01–3.93; *P*= 0.46), and fused level ≥3 (OR 3.69; 95% CI: 1.93–7.07; *P*=0.001) were independently associated with prolonged LOS (Table 5).

**Table 4:** Risk factors for postoperative major complications by univariate analysis and multivariate logistic regression analysis.

	Univariate analysis			Multivariate analysis	
Risk factors	*P*	No (215)	Yes (30)	*P*	Odds ratio (95% CI)
Female *n*/(%)	0.611	133 (61.9%)	20 (66.7%)		
Age (years)	0.167	79.9±3.4	79.0±3.5		
BMI (kg/m^2^)	0.739	24.8±3.5	25.0±3.9		
Charlson comorbidity index	0.015*				
0–1		136 (63.2%)	12 (40.0%)	0.040*	2.29 (1.04–5.08)
2 or more		79 (36.8%)	18 (60.0%)		
Current smoking	0.999	20 (9.3%)	3 (10.0%)		
Current drinking	0.999	12 (5.6%)	2 (6.7%)		
Frailty	0.023*	103 (47.9%)	21 (70.0%)	0.059	2.24 (0.97–5.18)
Dependence in ADL	0.779				
No		40 (18.6%)	4 (13.3%)		
Mild		122 (56.7%)	18 (60.0%)		
Moderate-severe		53 (24.7%)	8 (26.7%)		
Dependence in IADL	0.757				
No		46 (21.4%)	5 (16.7%)		
Mild		60 (27.9%)	10 (33.3%)		
Moderate-severe		109 (50.7%)	15 (50.0%)		
MNA-SF <12 points	0.566	54 (25.1%)	9 (30.0%)		
MMSE <24 points	0.727	151 (70.2%)	22 (73.3%)		
Diagnosis	0.871				
LSS		127 (59.1%)	19 (63.3%)		
DDD		52 (24.2%)	7 (23.3%)		
Lumbar spondylolisthesis		36 (16.7%)	4 (13.3%)		
Duration of symptoms (years)	0.250	5.7±7.1	7.4±9.2		
VAS-back at baseline	0.269	4.6±2.3	5.1±2.2		
VAS-leg at baseline	0.622	6.7±1.7	6.8±1.3		
ODI at baseline	0.162	47.9±18.6	52.9±15.1		
Surgery-related data					
ASA	0.542	2.7±0.5	2.7±0.5		
Number of fused segments	0.769				
1–2		156 (72.6%)	21 (70.0%)		
3–5		59 (27.4%)	9 (30.0%)		
Operative time (min)					
1–2 level fusion	0.354	194.3±58.4	207.0±63.3		
3–5 level fusion	0.292	273.2±76.1	301.4±58.7		
Estimated blood loss (ml)	0.261	377.1±360.8	299.3±302.4		

ADL, activities of daily living; ASA, American Society of Anesthesiologists; DDD, degenerative disc disease; IADL, instrumental activities of daily living; LSS, lumbar spine stenosis; MMSE, mini-mental state examination; MNA-SF, mini-nutritional assessment-short form; ODI, Oswestry Dability Index; VAS, visual analog scale.

* Represents for statistically different (*P*<0.05).

**Table 5:** Risk factors for prolonged length of hospital stay by univariate analysis and multivariate logistic regression analysis.

	Univariate analysis			Multivariate analysis	
Risk factors	*P*	No (184)	Yes (61)	*P*	Odds ratio (95% CI)
Female n/(%)	0.072	109 (59.2%)	44 (72.1%)	0.238	0.66 (0.33–1.32)
Age (y)	0.095	79.6±3.4	80.4±3.3	0.101	1.08 (0.99–1.18)
BMI (kg/m^2^)	0.235	24.6±3.6	25.3±3.6		
Charlson comorbidity index	0.389				
0 or 1		114 (61.9%)	34 (55.7%)		
2 or more		70 (38.1%)	27 (44.3%)		
Current smoking	0.382	19 (10.3%)	4 (6.6%)		
Current drinking	0.999	11 (5.9%)	3 (4.9%)		
Frailty	0.007*	84 (45.7%)	40 (65.6%)	0.041*	1.97 (1.03–3.78)
Dependence in ADL	0.136				
No		35 (19.0%)	9 (14.8%)		
Mild		109 (59.2%)	31 (50.8%)		
Moderate-severe		40 (21.8%)	21 (34.4%)		
Dependence in IADL	0.336				
No		42 (22.8%)	9 (14.8%)		
Mild		53 (28.8%)	17 (27.9%)		
Moderate-severe		89 (48.4%)	35 (57.4%)		
MNA-SF <12 points	0.005*	39 (21.2%)	24 (39.3%)	0.046*	1.94 (1.01–3.93)
MMSE <24 points	0.203	126 (68.5%)	47 (77.1%)		
Diagnosis	0.477				
LSS		112 (60.9%)	34 (55.7%)		
DDD		45 (24.5%)	14 (23.0%)		
Lumbar spondylolisthesis		27 (14.6%)	13 (21.3%)		
Duration of symptoms (years)	0.379	6.2±7.6	5.2±6.5		
VAS-back at baseline	0.150	4.5±2.2	5.0±2.5		
VAS-leg at baseline	0.404	6.7±1.6	6.5±1.8		
ODI at baseline	0.057	47.3±18.4	52.4±17.4	0.285	1.01 (0.99–1.03)
Surgery-related data					
ASA	0.752	2.7±0.5	2.74±0.4		
Number of fused segments	<0.001*				
1–2		146 (79.4%)	31 (50.8%)		
3–5		38 (20.6%)	30 (49.2%)	0.001*	3.69 (1.93–7.07)
Operative time (min)					
1–2 level fusion	0.115	191.8±59.5	207.9±56.3		
3–5 level fusion	0.612	274.8±73.1	286.9±81.9		
Estimated blood loss (ml)	0.865	369.8±361.3	360.8±335.9		

ADL, activities of daily living; ASA, American Society of Anesthesiologists; DDD, degenerative disc disease; IADL, instrumental activities of daily living; LSS, lumbar spine stenosis; MMSE, mini-mental state examination; MNA-SF, mini-nutritional assessment-short form; ODI, Oswestry Dability Index; VAS, visual analog scale.

* Represents for statistically different (*P*<0.05).

## Discussion

Through implementing ERAS protocols, patients undergoing lumbar fusion often experience accelerated recovery, typically accompanied by lower morbidity and mortality rates^29^. With an increasing number of oldest-old patients, the value of implementing ERAS protocols in the frail patients, particularly those aged 75 years and above, has been questioned^11,30^. This prospective study examined the association between Fried frailty and post-ERAS outcomes in older patients undergoing scheduled TLIF surgery. Our findings suggest that Fried frailty, MNA-SF <12 points, and fused level ≥3 were independent predictors for increased length of stay, while CCI ≥2 was the sole predictor of 90-day major complications.

Frailty is characterized by age-related conditions leading to decreased energy and muscle strength, weight loss, malnutrition, and reduced mobility, thus increasing an individual’s vulnerability to dependency or death^13^. In our study cohort, 124 (50.6%) patients were frail, which is more than in previous studies^17,31^. The prevalence of frailty and prefrailty increased with age with peak prevalence in the oldest age group^32^. We did not find an association between Fried frailty and greater age, possibly because of our selection criteria. We included only patients 75 and older and excluded patients with inoperable disease. However, the association between greater age and impaired ADL remained in the older population with lumbar spinal disorders. This observation suggests that the relationship between age and ADL, as well as IADL, is relatively stable and less influenced by the diverse characteristics of the study sample. Malnutrition, muscle weakness, and comorbidities are important risk factors and key evaluation indicators of frailty^32^. Sarcopenia is a progressive and generalized skeletal muscle disorder that involves the accelerated loss of muscle mass and function that is associated with functional decline and impaired activities of daily living (ADL)^33^. Our correlation analysis revealed a significant association between frailty and impaired activities of daily living, as well as malnutrition. This finding provides some support for the implementation of multimodal antifrailty intervention, including nutritional support, chronic diseases management, and physical intervention.

The prevalence of frailty is increasing among individuals with lumbar spinal disorders, primarily driven by a decline in mobility and an increase in dependence on daily activities^34^. Frailty assessment is a useful preoperative risk screening instrument, particularly in the context of an aging population, and its significance is rapidly growing. A recent systematic review and meta-analysis of 17 studies on patients undergoing spinal surgery revealed that preoperative frailty was associated with a two-fold more significant risk of mortality, prolonged LOS, major complications, and two-fold more significant risk of nonhome discharge^35^. Although many instruments such as clinical frailty scales, FRAIL questionnaire, Modified Frailty Index (mFI), and Edmonton Frailty Scale have been developed in recent years, the mFI and Fried Frailty Scale remain the most frequently employed tools in clinical practice^15^. Previous studies reported that the mFI-5 and mFI-11 were both associated with postoperative complications with comparable predictive power^36^. The mFI is a deficit accumulation measure of frailty, which mainly focusing on pre-existing comorbidities. Charlson comorbidity index is a convenient tool to assess comorbidity severity and predict mortality risk of surgical patients^37^. The current study found that high-risk comorbidity (CCI ≥2), but not advanced age or Fried frailty, was an independent prognostic factor of major complications. Future studies should aim to evaluate the predictive value of CCI and mFI-5 in predicting major or overall complications among patients undergoing complex spine surgery or those with advanced age.

Another significant result of our study is that Fried frailty was significantly associated with prolonged LOS. Previous research has demonstrated that preoperative frailty is associated with postoperative complications and poor recovery following elective procedures^38^. Our study suggests that even with the implementation of ERAS protocols, Fried frailty remains a significant predictor of poor postoperative recovery. While ERAS optimizes the perioperative management of TLIF surgery, frail patients still face considerable challenges in restoring metabolic balance, physical rehabilitation, and regaining independence after surgery^30^. Identifying modifiable factors that significantly correlate with frailty and substantially impact postoperative outcomes is fundamental in establishing operational management programs for older patients. We found that an MNA-SF score below 12 points, which serves as a cutoff value for the risk of malnutrition, was a predictor of prolonged LOS. MNA-SF is a screening scale used to assess nutritional status; however, it also includes queries regarding other geriatric issues, including cognitive impairment, mobility, acute disease or psychological stress, weight loss, and food intake, which can not only define malnutrition but can also provide information regarding physical function decline^23^. Surgical stress can induce a catabolic state, where the body breaks down tissues, especially proteins, to meet the increased energy demands and support the healing process. This catabolic state can result in muscle protein breakdown, leading to muscle wasting and potentially compromising the overall recovery process^39,40^. Malnutrition and decreased physiological reserve hinder the body’s ability to maintain homeostasis during physiological stress. Nutritional intervention before, during, and after surgery plays a crucial role in optimizing nutrient stores and supporting the body’s ability to cope with the stress of surgery. Adequate nutrition is essential for various physiological processes, including immune function, tissue repair, and maintenance of muscle mass^41^. Frail patients have a predisposition to poor tolerance for early rehabilitation and home exercise, leading to an increasing dependency on institutional care compared to robust elderly patients. Balance and strength training can also prepare the patient for postoperative activities, emphasizing the strengthening of muscles that are most utilized during the recovery period^42^.

Prehabilitation comprises multidisciplinary healthcare interventions, including exercise, nutritional optimization, and psychological preparation, which aim to strengthen physiological reserve and enhance functional capacity^43^. Several meta-analyses and randomized trials on multimodal prehabilitation have reported clinical and functional benefits, yet only a few studies have reported using prehabilitation in an ERAS setting^11^. A retrospective analysis on colorectal cancer surgery revealed that ERAS patients who underwent multimodal prehabilitation maintained their fat-free mass at 8 weeks postsurgery, in comparison to ERAS patients who received only rehabilitation^44^. Another prospective study demonstrated that twice as many prehabilitated patients recovered their functional walking capacity, as defined by the 6 min walk test, at 8 weeks after surgery compared to the patients who received ERAS care alone. These findings contribute valuable insights into the efficacy of comprehensive perioperative strategies, emphasizing the importance of integrating prehabilitation within ERAS protocols for improving postoperative outcomes in colorectal cancer surgery. In practice, the specific exercise regimen varies significantly between studies, concerning exercise type and duration (from 2 to 12 weeks), and there is limited knowledge regarding the dose-response relationship^44^. While our ERAS program included short-term inpatient preoperative prehabilitation, it was observed that prefrail patients exhibited a higher incidence of poor postoperative recovery. Integration of well-established prehabilitation, which enhances tolerance to surgical stress, with ERAS care, which minimizes surgical stress, has the potential to promote surgical resilience^30^.

This is a prospective cohort study of patients aged ≥75 years who underwent enhanced recovery after lumbar fusion surgery for degenerative spine disease. Frail patients were compared with non/prefrail patients regarding the incidences of postoperative AEs. The principal strength of this study is the use of high-quality data from a prospective observational cohort. This yielded high completion rates for most outcome measures and data that reflect ‘real-world’ practice. Moreover, the average age of patients in our study is older than previously mentioned. Our study is also strengthened by implementing clinical outcomes and follow‐up patient-reported outcomes, which have not been thoroughly evaluated in previous studies.

The present study has several limitations. First, this was a single-center exploratory study. The sample sizes were chosen based on previous experience and similar studies due to the lack of data on older ERAS patients. Our sample size was 253, and therefore, it met the minimum requirements. Nevertheless, the sample size of the current study is relatively small. A multicenter study with a large sample size may increase the generalizability of the study results. Second, due to the nature of the prospective study, we cannot include frail patients who received conventional care. A previous retrospective analysis of patients >65 years of age undergoing open TLIF revealed that ERAS significantly improves the return of physiologic function and length of stay in frail patients compared to non-ERAS frail patients^17^. Our study suggests that ERAS alone may not be sufficient for frail older patients, who represent the most vulnerable population in in surgical practice. Third, given the data distribution, nonfrail and prefrail patients were combined into one group. The proportion of robust individuals with lumbar disorders is relatively low among the oldest-old patients. Fourth, we only employed the Fried frailty scale as a preoperative screening tool; hence, we cannot compare the predictive values of the Fried frailty scale with other instruments, such as the mFI-5 and FRAIL scale^15^. Implementing more objective measures like muscle mass and grip strength will also be helpful for frailty assessment. Lastly, to avoid bias resulting from different surgical procedure, we only included patients undergoing TLIF surgery. High-quality multicenter studies may better elucidate the role of ERAS in a broader population of spine surgical patients.

## Conclusion

Preoperative frailty, as assessed by the Fried frailty scale, was associated with increased 90-day AEs rates following scheduled TLIF surgery in post-ERAS patients aged ≥75 years. A higher CCI grade was an independent predictor for 90-day major complications, while Fried frailty and MNA-SF scores <12 predicted poor postoperative recovery. Continued research and multidisciplinary collaboration will be essential to refine and optimize protocols for surgical care in frail older adults.

## Ethical approval

This study was approved by the Ethical review committee of Xuanwu Hospital, Capital Medical University (IRB#2018-086).

## Consent

All included patients filed informed consent for data use.

## Sources of funding:

This research received grant from Seed Program of Xuanwu Hospital of Capital Medical University (No. YC20220103), National Key Research and Development Program of China (No. 2020YFC2004900), and Post-subsidy funds for National Clinical Research Center, Ministry of Science and Technology of China (No. 303-01-001-0272-05). The funding organization did not participate in the design and conduct of the study; in the collection, analysis, and interpretation of the data; or in the preparation, review, or approval of the manuscript.

## Author contribution

S.-K.W. and S.-B.L.: mainly contributed to the conception of the study and wrote the manuscript; Q.-J.W. and X.-L.C.: made an important contribution to the revision of the manuscript. Other authors mainly performed the data collection and data analyses.

## Conflicts of interest disclosure

The authors declare that they have no conflicts of interest.

## Research registration unique identifying number (UIN)

Chinese clinical trial (trial no. ChiCTR1800020363).

## Guarantor

Shuaikang Wang, Department of Orthopedics, Xuanwu Hospital of Capital Medical University, 45 Changchun Street, Xicheng, Beijing 100053, People’s Republic of China, E-mail: jackwangDR@163.com. Shibao Lu, Department of Orthopedics, Xuanwu Hospital of Capital Medical University, 45 Changchun Street, Xicheng, Beijing 100053, People’s Republic of China, E-mail: shibaolu@xwh.ccmu.edu.cn.

## Data availability statement

All data and materials can be obtained by mail of the corresponding author.

## Provenance and peer review

Not commissioned, externally peer-reviewed.
